# Intraocular Inflammation with faricimab: insights from Manufacturer and User Facility Device Experience (MAUDE) database

**DOI:** 10.1038/s41433-024-03079-0

**Published:** 2024-04-24

**Authors:** Ibraheem M. Alkhawaldeh, Hashem Abu Serhan

**Affiliations:** 1 Faculty of Medicine, Mutah University, Al-Karak, Jordan; 2 Department of Ophthalmology, Hamad Medical Corporation, Doha, Qatar

**Keywords:** Risk factors, Retinal diseases, Education

Since its introduction in 2019, brolucizumab’s safety has been called into question due to numerous reports of IOI following intravitreal injections [1]. In 2020, post-marketing data showed that there was a confirmed safety alarm of severe ocular adverse events such as retinal vasculitis, and retinal vascular occlusion that may lead to severe visual acuity loss. These events were reported at rates of 3.61 and 2.35 per 10,000 injections, respectively [1]. New concerns have been raised by Thangamathesvaran et al. on intraocular inflammation (IOI) after intravitreal faricimab (Vabysmo) [2]. Therefore, investigating underlying risk factors for IOI development following faricimab would enhance our understanding of this safety concern and inform clinical decision-making.

Many hypothesized causes, including patient susceptibility, the manufacturing process, and agent characteristics, are responsible for the IOI development after intravitreal injections [2]. The manufacturing process of faricimab may induce IOI through protein aggregates of the injection that could release silicone oil from syringes, causing IOI [3]. The Manufacturer and User Facility Device Experience (MAUDE) database of the US Food and Drug Administration (FDA) is a publicly available database representing reports of adverse events involving medical devices [4]. Herein, we aimed to share that we observed a growing number of monthly reports about similar manufacturing concerns related to faricimab which may support that handling and delivery problems could be a main risk factor of IOI.

A targeted search in MAUDE database using the terms “Faricimab” and “Vabysmo” yielded 8 reports, with the initial one recorded on October 16, 2023 (Table 1). Among these, seven reports highlighted concerns related to sterility and contamination issues associated with the needle filter. These issues included the presence of foreign substances, a faulty filter needle that fractured during vial insertion, leading to drug leakage, a complaint about a fiber wrapped around the filter needle, and a case where the stopper of the filter needle was found positioned ‘way up the middle’ of the filter needle. Furthermore, a separate incident was reported where the needle fell apart before administration. Notably, most reports were identified before any ocular manifestations. In contrast, two reports highlighted patients experiencing IOI; one was endophthalmitis and the other was not specified.

**Table 1:** Adverse events of faricimab reported from MAUDE database.

Report number	Event date	Event type	Manufacturer	Brand name	Device problem	Patient problem	Event description
1911916-2024-00006	2024/01/04	Malfunction	BECTON DICKINSON	NEEDLE FILTER BLUNT FILL 18×1-1/2	Device Contamination with Chemical or Other Material	No Clinical Signs, Symptoms or Conditions	Roche received a market complaint indicating that a foreign substance was found in the needle when it was inserted to administer the product to a patient. The product was not used. Roche considers this a high-risk and potentially critical complaint due to the possibility of contaminating a sterile product. One photo was provided for evaluation, showing a magnified needle assembly with no packaging blister or plastic shield. The needle has a dark-colored particle adhered close to the dome, with no other observed defects.
1911916-2023-00929	2023/12/18	Injury	BECTON DICKINSON	NEEDLE FILTER BLUNT FILL 18×1-1/2	Adverse Event Without Identified Device or Use Problem	Endophthalmitis	Roche has received a complaint stating that the patient suffered infectious endophthalmitis after the administration of one unit of VABYSMO.
1911916-2023-00926	2023/12/18	Malfunction	BECTON DICKINSON	NEEDLE FILTER BLUNT FILL 18×1-1/2	Device Contamination with Chemical or Other Material	No Clinical Signs, Symptoms or Conditions	Roche has received a complaint where a purchasing supervisor, reporting on behalf of a technician, observed a foreign matter in the syringe after drawing up the dose from a VABYSMO 6MG vial using the supplied filter needle at the HCP office prior to administration.
1911916-2023-00904	2023/12/04	Malfunction	BECTON DICKINSON	NEEDLE FILTER BLUNT FILL 18×1-1/2	Break	No Clinical Signs, Symptoms or Conditions	A complaint was received where a Healthcare Professional (HCP) reported that VABYSMO had a faulty filter needle that fractured upon insertion into the vial, causing the drug to leak out. The HCP did not observe an initial defect in the filter needle, and the leakage occurred as the HCP was drawing up the contents.
1911916-2023-00885	2023/11/17	Malfunction	BECTON DICKINSON	NEEDLE FILTER BLUNT FILL 18×1-1/2	Device Contamination with Chemical or Other Material	No Clinical Signs, Symptoms or Conditions	A needle-related issue involving foreign matter, specifically a fiber wrapped around the filter needle, was reported by an HCP office through the PQC mailbox for the product VABYSMO (6 mg vial). Limited information is available, and the physical sample’s status is unknown. The complaint is considered potentially critical due to the risk of contamination of a sterile product, necessitating an expedited investigation. The report was confirmed through photo analysis.
1911916-2023-00855	2023/10/30 4:00:00	Injury	BECTON DICKINSON	NEEDLE FILTER BLUNT FILL 18×1-1/2	Adverse Event Without Identified Device or Use Problem	Unspecified Eye/Vision Problem	A Therapeutic Area Manager reported a potential adverse event on behalf of a Healthcare Professional (HCP). Approximately 3 months ago, on (B)(6) 2023, the patient developed vision issues that may have been related to a sterility issue with the product VABYSMO.
1911916-2023-00814	2023/10/19 4:00:00	Malfunction	BECTON DICKINSON	NEEDLE FILTER BLUNT FILL 18×1-1/2	Device Contamination with Chemical or Other Material	No Clinical Signs, Symptoms or Conditions	A Genentech Therapeutic Area Manager reported a complaint on behalf of a Healthcare Professional (HCP): “When drawing up medication from VABYSMO, the tech noticed that the stopper of the filter needle was ‘way up the middle’ of the filter needle.” The issue is identified as device contamination with chemical or other material.
1911916-2023-00804	2023/10/16 4:00:00	Malfunction	BECTON DICKINSON	NEEDLE FILTER BLUNT FILL 18×1-1/2	Loss of or Failure to Bond	No Clinical Signs, Symptoms or Conditions	A complaint was reported wherein the Healthcare Professional (HCP) office contact stated that the needle from one VABYSMO 6MG vial fell apart before administration in an HCP setting.

In conclusion, manufacturer and handling issues may overstate the challenges and adverse events following faricimab. Prospective monitoring and surveillance system reporting are required in the future to discover potential concerns before they cause health issues or repercussions.

## Data Availability

All the data used in this study are available within the article.
